# Racial Disparities in Comorbidity Patterns of Early-Onset Liver Cancer: A Machine Learning Analysis

**DOI:** 10.1177/10732748251363687

**Published:** 2025-07-30

**Authors:** Bingya Ma, Kai Zheng, Fa-Chyi Lee, Yunxia Lu

**Affiliations:** 1 Department of Epidemiology and Biostatistics, University of California Irvine, Irvine, CA, USA; 2 Department of Informatics, Donald Bren School of Information and Computer Science, University of California Irvine, Irvine, CA, USA; 3 Department of Emergency Medicine, School of Medicine, University of California Irvine, Irvine, CA, USA; 4 Division of Hematology/Oncology, Department of Medicine, University of California Irvine, Orange, CA, USA; 5 Department of Population Health and Disease Prevention, University of California Irvine, Irvine, CA, USA

**Keywords:** racial disparities, liver cancer, machine learning, comorbidity, SHAP

## Abstract

**Introduction:**

The incidence of early-onset liver cancer (EOLC) has been increasing in many countries, yet evidence on its etiology remains limited, particularly outside the Asian population. This case-control study explores the comorbidity patterns of EOLC and develops race/ethnicity-specific machine learning (ML) models to predict liver cancer risk.

**Methods:**

We included patients diagnosed with primary liver cancer between ages 18 and 49 from the University of California Health Data Warehouse, matching each patient with five controls. ML classification methods, including decision trees, random forests, logistic regression, XGBoost, and LightGBM, were used to assess liver cancer risk based on demographics and comorbidities. Model performance was evaluated using F1 scores, and SHapley Additive exPlanations (SHAP) was applied to identify the most influential comorbidities within each racial group.

**Results:**

A total of 1574 patients and 7870 controls were identified. Asian and Pacific Islanders (API) had significantly higher rates of Hepatitis B virus (HBV) infection, while Hispanics had higher prevalences of cirrhosis, hypertension, diabetes, and Hepatitis C virus (HCV) infection. Whites showed higher rates of anxiety, asthma, hypothyroidism, and cholangitis. Race/ethnicity-specific models for API (F1 score = 0.77, AUC = 0.90) and Hispanics (F1 score = 0.77, AUC = 0.92) outperformed the model for Whites (F1 score = 0.64, AUC = 0.87) in the validation dataset. The SHAP results indicated that HBV infection was the dominant comorbidity for API, and HCV and metabolic disorders were notable among Hispanics. In contrast, the White population showed a broader and less concentrated comorbidity pattern.

**Conclusions:**

Our study highlights significant racial disparities in comorbidity patterns for early-onset liver cancer, demonstrating the potential of ML models to identify high-risk populations and inform targeted prevention strategies.

## Introduction

Primary liver cancer, mainly including hepatocellular carcinoma (HCC) and intrahepatic cholangiocarcinoma (ICC), is the sixth most frequently diagnosed cancer worldwide.^1^ Liver cancer exhibits distinct incidence rates across different populations, with a range of factors contributing to its development within specific racial groups.^2-4^ From 1998 to 2015, the incidence rates of liver and intrahepatic bile duct cancer in the U.S. increased, peaking with an annual percent change (APC) of 4.5%. This was followed by a stabilization period, with a marginal APC of 0.3% from 2015 to 2021.^5^

Early-onset liver cancer (EOLC) is often defined as liver cancer diagnosed in individuals younger than 50 years of age. According to data from the Surveillance, Epidemiology, and End Results registries, the incidence of early-onset HCC decreased in the United States from 2010 to 2019, whereas the incidence of early-onset ICC increased during the same period.^6^ Additionally, the incidence of EOLC has been rising in regions such as East Asia, Australia, Slovakia, and Uganda.^7,8^ The reasons for these different incidence patterns across different populations are unknown.

Clinical characteristics of EOLC differ from the liver cancer diagnosed at older ages. For example, compared to 80-90% of cirrhosis in all HCC patients, only 12.7-33.3% of young-onset HCC cases had liver cirrhosis.^8,9^ In addition, young HCC patients had a significantly higher rate of Hepatitis B surface antigen (HBsAg) positivity, better liver function, and a more advanced tumor stage at diagnosis compared with the older group.^9^ This indicates a distinct precancerous disease pattern in EOLC. So far, a few studies have identified risk factors and precancerous diseases for EOLC, including male gender, Hepatitis B virus (HBV), smoking, family history, and previous chronic liver disease.^10-14^ However, the previous studies were primarily conducted among the Asian populations, and research evidence in other races is sparse, leaving gaps in understanding the etiologies of EOLC.^10-14^ A deeper understanding of comorbidity patterns may help identify risk factors and high-risk populations across different racial groups.

In virtue of the medical record data from the University of California Health Data Warehouse (UCHDW), we have an opportunity to initiate a retrospective case-control study to examine the comorbidity patterns among racial/ethnic groups. The UCHDW is a research data warehouse aggregating electronic health records (EHR) data from 6 UC Health campuses (Davis, San Francisco, Los Angeles, Riverside, Irvine, and San Diego. It contains high-quality clinical information, including diagnoses, lab tests, prescriptions, and more. In addition, advances in analytical methods, especially the development of machine learning (ML) approaches, make it possible to analyze large-scale, real-world EHR data.^15^ Different from traditional statistical models, which rely on strict assumptions regarding data distributions and face challenges with missing data, ML techniques are more flexible and better suited to handle complex and incomplete datasets.

Therefore, we conducted this study to leverage the power of both UCHDW and ML to examine the patterns of comorbidities across different races, develop race/ethnicity-specific ML models to predict liver cancer, and identify the most important comorbidities in each racial/ethnic group. The goal is to identify high-risk groups and promote targeted prevention and control of liver cancer among younger populations.

## Methods

### Study Population

We initiated a matched case-control study based on the UCHDW, which contained de-identified data on over 10 million patients dating back to 2012.^16^ The dataset reflects California's diverse population, including substantial representation across various racial and ethnic groups. The UCHDW is encoded according to the Observational Medical Outcomes Partnership Common Data Model (OMOP CDM), which standardizes data structure and coding terminologies to facilitate data sharing across healthcare institutions.^17^ Patients diagnosed with primary liver cancer (SNOMED code 95214007, ICD-O-3 and ICD-10 code C22, and/or ICD-9 code 155), aged 18 to 49 years, were included. We further excluded codes for secondary cancer, metastasis, and hepatoblastoma, the latter having a distinct etiology as the most common childhood liver cancer. Then, we identified standard concept IDs and retrieved relevant descendant codes to capture all patients with liver cancer. In addition, we excluded patients with prior diagnoses of other cancer types to minimize the inclusion of metastatic liver cancer cases. We also removed individuals with missing demographic information (e.g., age, gender, race/ethnicity) from the analysis. To enhance the completeness of the patient cohort, we only included patients with two or more hospital visits in the UCHDW, which provides a more reliable representation of disease prevalence by reducing the likelihood of including patients with incomplete or sporadic data.^18^ For each liver cancer case, we identified all eligible controls matched on sex, race, and birth year—demographic factors known to influence liver cancer risk—to ensure balanced representation across racial and ethnic groups. The further eligibility of controls included no history of any cancers, having at least two hospital visits, and the observation period encompassing the diagnosis date of the corresponding cases, which ensured comparable exposure windows. In the pool of eligible controls for each case, we applied a random sampling approach using PySpark window functions with randomized row ordering to select five controls. A 1:5 case-to-control ratio was chosen to maximize the information gained from the limited number of cases, enhance statistical efficiency, and improve model stability.^19^ The index date for each case was defined as the first date of diagnosis of primary liver cancer in the UCHDW, and the corresponding index date for the five matched controls was the same as the index date of the matched case.

This study uses a limited data set (LDS) version of the UCHDW that has all patient identifiers removed except for service dates and year of birth. The use of the LDS UCHDW in secure research computing enclaves was approved jointly by the Institutional Review Boards (IRB) of all UC Health campuses as non-human subject research. The reporting of this study conforms to STROBE guidelines.^20^

### Data Retrieval

Patients were grouped based on self-reported race and ethnicity groups, identified using the following SNOMED codes: non-Hispanic Asian and Pacific Islanders (API) (race: 8515, 8557; ethnicity: 38003564), Hispanic (ethnicity: 38003563), non-Hispanic White (race: 8527; ethnicity: 38003564), and other or unknown races, the latter contained liver cancer cases in non-Hispanic Black, American Indian/Alaska Native, and other racial/ethnic groups and those with unknown races/ethnicities. Further demographic information of year of birth, gender, and socioeconomic status (SES) was identified from the UCHDW as follows. Age was calculated by subtracting the year of birth from the year of the first liver cancer diagnosis (the index date) for each case-control matched group. SES was assessed using the Area Deprivation Index (ADI) scores, which combine various socioeconomic indicators at the census block group level—such as income, education, employment, and housing quality—to quantify neighborhood-level socioeconomic disadvantage.^21^ It ranks from least to most deprived and is divided into deciles represented by integers from 1 to 10.^22^ Missing ADI scores were imputed using MissForest, an ML algorithm based on random forests for handling missing data. Compared to traditional imputation methods, MissForest can handle both continuous and categorical variables and capture complex relationships and interactions between variables, and it does not rely on assumptions about the underlying data distribution.^23^

Comorbidities were defined as preexisting conditions diagnosed before or at the onset of liver cancer, with symptoms or complications of certain diseases excluded, as they are signs or adverse events associated with comorbidities.^24^ To ensure adequate statistical power, we included comorbidities with a prevalence greater than 1% among cases or those previously reported as risk factors for liver cancer or liver diseases. For example, Crohn’s disease, one of the inflammatory bowel diseases, and colon polyps are closely associated with several liver and biliary diseases.^25-27^ Although the prevalence of these conditions was slightly lower than 1%, we included them in this study. Patients were identified as having specific comorbidities if they had corresponding diagnoses (identified by SNOMED or ICD codes), abnormal lab test results, or records of specific medications before or on the index date. Detailed criteria for each comorbidity are described in Table S1. Comorbidities were further classified by subtype of the diseases and whether the condition had begun within or more than one year before the index date. The comorbidity classification categories are provided in Table S2.

### Statistical Analysis

We conducted descriptive statistics to describe the demographic characteristics of cases and controls. Numeric variables were displayed as medians and interquartile ranges (IQRs); categorical variables were presented as frequencies and proportions. The ten most prevalent comorbidities, along with their prevalence in the corresponding controls, are listed in Table 1. Odds ratios (ORs) with 95% confidence intervals (CIs) were estimated to measure the association between each comorbidity and the risks of liver cancer.

**Table 1.** Demographic Characteristics of Cases and Controls

Variable	Cases (N = 1574)	Controls (N = 7870)
Age, median (IQR), years	43 (35-47)	43 (35-47)
Age group (years)		
18-29	205 (13.0%)	1025 (13.0%)
30-39	403 (25.6%)	2015 (25.6%)
40-49	966 (61.4%)	4830 (61.4%)
Gender		
Male	931 (59.1%)	4655 (59.1%)
Female	643 (40.9%)	3215 (40.9%)
Race		
Asian and pacific islander	349 (22.2%)	1745 (22.2%)
Hispanics	446 (28.3%)	2230 (28.3%)
White	466 (29.6%)	2330 (29.6%)
Other/Unknown	313 (19.9%)	1565 (19.9%)
ADI, median (IQR)	5 (3-7)	4 (2-7)
ADI group		
1-3	461 (29.3%)	3171 (40.3%)
4-7	628 (39.9%)	2952 (37.5%)
8-10	344 (21.9%)	1388 (17.6%)
Missing	141 (9.0%)	359 (4.6%)

*Note:* ADI, Area Deprivation Index; IQR, interquartile range.

In this study, we leveraged the AutoML modules provided on the Databricks Platform (Databricks, Inc, San Francisco, CA) to train and construct race/ethnicity-specific predictive ML models for the risk of EOLC using information on age, sex, ADI scores, and comorbidities. The dataset was randomly split into training (60%), validation (20%), and test (20%) datasets for model development, tuning, and evaluation, respectively. Classification methods utilized by Databricks AutoML, including decision trees, random forests, logistic regression, XGBoost, and LightGBM, were employed. The effectiveness of the selected models in predicting cancer risk has been demonstrated in previous studies.^28,29^ To ensure comparability, all models were trained and evaluated using the same predefined set of features. Feature selection and hyperparameter tuning were automatically managed by Databricks AutoML for each model. ​Databricks facilitated the hyperparameter tuning by integrating distributed optimization libraries like Optuna and Ray Tune with MLflow for tracking, enabling scalable and efficient model selection across clusters. AutoML also addressed class imbalance in the dataset by down-sampling the majority classes and applying class weights when an imbalance was detected.^30^ The F1 scores for all classification methods are presented in Table S3, and the method achieving the highest F1 score on the validation dataset was selected for final evaluation and reporting. The F1 score was used as the primary evaluation metric due to its effectiveness in balancing precision and recall, particularly in imbalanced datasets in which each case was matched with five controls.^31^ The area under the curve (AUC) values were also reported to evaluate the models’ performance. A summary of the model training and validation process is provided in Table S4. We also used SHapley Additive exPlanations (SHAP) to interpret the machine learning model outputs. SHAP is a unified approach based on Shapley values from cooperative game theory that quantifies the average marginal contribution of each feature across all possible feature combinations. This allows for transparent and consistent interpretation of how each comorbidity influences the predicted liver cancer risk while accounting for complex interactions with other variables.^32^ Mean SHAP values were calculated to summarize the overall contribution of each feature to model prediction results using the SHAP package. A summary of the SHAP method, including its mathematical foundation, model compatibility, and application in this study, is provided in Table S5.

Subgroup analysis among patients with HCC diagnosis, the major histological type of primary liver cancer, was further conducted. For comparison purposes, a similar data extraction and analysis process was applied to analyze the comorbidity patterns among late-onset liver cancer (LOLC) patients who were first diagnosed at age 50 years or older, along with five matched controls. All data extractions and analyses were performed on Databricks via Amazon Web Services (AWS, Amazon.com, Inc, Seattle, WA) 13.3 LTS, SQL 3.5.1, and Python 3.10.12.

## Results

Among 9,447,655 patients included in the UCHDW dataset between Jan 1, 2012, and August 5, 2024, we identified 2288 patients who were first diagnosed with liver cancer between the ages of 18 and 49 years. Of these, we excluded 605 patients with a diagnosis of another cancer before liver cancer, one patient with unknown gender information, 105 patients with fewer than two visits, and three patients matched with only two controls. Finally, 1574 EOLC patients remained in the analysis, and 7870 matched controls were identified. The process of data collection is shown in Figure S1.

The baseline demographic characteristics among cases and controls are shown in Table 1. The median age of the study population was 43 years (IQR 35-47 years), and 59.1% were male. Among the liver cancer patients, 22.2% were API, 28.3% were Hispanic, 29.6% were White, and 19.9% were of other or unknown races. The median ADI was 5 (IQR 3-7) among cases and 4 (IQR 2-7) among controls. We identified 31 comorbidities in the analyses, including liver diseases (HBV infection, Hepatitis C virus (HCV) infection, cirrhosis, steatosis of the liver, autoimmune liver disease), biliary diseases (gallstone, cholesterolosis of the gallbladder, cholangitis), metabolic disorders (diabetes, hyperlipidemia, hypertension), mental health disorders (anxiety, depressive disorder), gastrointestinal diseases (gastroesophageal reflux disease or peptic ulcer, ulcerative colitis, Crohn’s disease, polyp of the large intestine), renal conditions (chronic kidney disease, kidney stone), substance use disorders (alcohol dependence, nicotine dependence), respiratory or allergic diseases (asthma, obstructive sleep apnea, allergic rhinitis), cardiovascular disease (congenital heart disease, coronary arteriosclerosis), and other health conditions (vitamin D deficiency, hypothyroidism, anemia, Human Immunodeficiency Virus [HIV] infection). Comorbidities included in the LOLC models were different due to the different prevalence of the diseases in the older patients. For example, congenital heart disease, HIV infection, ulcerative colitis, and Crohn’s disease were not included in the LOLC models because of the extremely low prevalence. In contrast, cerebrovascular disease, myocardial infarction, peripheral vascular disease, chronic obstructive pulmonary disease, prostatic hyperplasia, gout, osteoarthritis, osteoporosis, cataract, and diverticular disease of the colon were included in the model for the LOLC.

Table 2 presents the results for the top comorbidities among the cases of EOLC and LOLC by racial groups. In the API group, HBV infection exhibited a high prevalence (47.3%) and the strongest association with EOLC (OR = 64.3, 95% CI: 40.8-101.3). Hypertension, cirrhosis, GERD/peptic ulcer, diabetes, HCV infection, and hyperlipidemia were common comorbidities across all racial groups, with all conditions positively associated with EOLC except hyperlipidemia, which might be due to the impaired hepatic function in lipid synthesis among patients with liver diseases.^33^ Additionally, anemia (OR = 1.8, 95%CI: 1.3-2.5), alcohol dependence (OR = 9.0, 95%CI: 5.7-14.2), anxiety (OR = 1.8, 95%CI: 1.3-2.5), and steatosis of the liver (OR = 6.4, 95%CI: 4.2-9.9) in Hispanics were positively associated with EOLC, with notable prevalence higher than 10% in the cases. Among White patients, anxiety (OR = 1.7, 95%CI: 1.3-2.3), asthma (OR = 1.6, 95%CI: 1.2-2.2), and hypothyroidism (OR = 1.6, 95%CI: 1.1-2.3) were significantly associated with EOLC. Cholangitis was prevalent among White patients and was strongly associated with EOLC (OR = 68.9, 95% CI: 21.2-224.1). For LOLC cases, HCV infection, alcohol dependence, asthma, hypothyroidism, and metabolic syndrome-related comorbidities, such as hypertension, hyperlipidemia, and diabetes, were more common. In contrast, HBV infection and anxiety were comparatively less prevalent in the LOLC group, suggesting a distinct risk profile for comorbidities between early- and late-onset liver cancer cases.

**Table 2.** Top Ten Comorbidities With Highest Prevalence Among Early- and Late-Onset Liver Cancer Cases by Race

Race	Early-onset liver cancer				Late-onset liver cancer			
	Comorbidity	Cases (n, %)	Controls (n, %)	OR (95% CI)	Comorbidity	Cases (n, %)	Controls (n, %)	OR (95% CI)
Asian and pacific islander	HBV	165 (47.3%)	24 (1.4%)	64.3 (40.8-101.3)	Hypertension	2020 (64.1%)	7379 (46.8%)	2.0 (1.9-2.2)
	Hypertension	95 (27.2%)	306 (17.5%)	1.8 (1.3-2.3)	Diabetes	1390 (44.1%)	5490 (34.8%)	1.5 (1.4-1.6)
	Cirrhosis	77 (22.1%)	8 (0.5%)	61.5 (29.3-128.7)	Hyperlipidemia	1208 (38.3%)	6937 (44.0%)	0.8 (0.7-0.9)
	Hyperlipidemia	72 (20.6%)	415 (23.8%)	0.8 (0.6-1.1)	GERD/Peptic ulcer	1069 (33.9%)	3115 (19.8%)	2.1 (1.9-2.3)
	GERD/Peptic ulcer	71 (20.3%)	176 (10.1%)	2.3 (1.7-3.1)	HBV	901 (28.6%)	319 (2.0%)	19.4 (16.9-22.2)
	Diabetes	69 (19.8%)	314 (18.0%)	1.1 (0.8-1.5)	Cirrhosis	877 (27.8%)	116 (0.7%)	52.0 (42.6-63.4)
	Steatosis of liver	25 (7.2%)	24 (1.4%)	5.5 (3.1-9.8)	HCV	412 (13.1%)	86 (0.6%)	27.4 (21.6-34.7)
	Vitamin D Deficiency	21 (6.0%)	120 (6.9%)	0.9 (0.5-1.4)	Asthma	269 (8.5%)	1457 (9.2%)	0.9 (0.8-1.0)
	HCV	18 (5.2%)	0 (0.0%)	Undefined^a^	Kidney Diseases	235 (7.5%)	1099 (7.0%)	1.1 (0.9-1.2)
	Asthma	17 (4.9%)	132 (7.6%)	0.6 (0.4-1.1)	Hypothyroidism	211 (6.7%)	1042 (6.6%)	1.0 (0.9-1.2)
Hispanic	Hypertension	251 (56.3%)	502 (22.5%)	4.4 (3.6-5.5)	Hypertension	2951 (74.6%)	9437 (47.7%)	3.2 (3.0-3.5)
	Cirrhosis	218 (48.9%)	17 (0.8%)	124.5 (74.6-207.7)	Cirrhosis	2284 (57.7%)	428 (2.2%)	61.7 (55.0-69.2)
	GERD/Peptic ulcer	169 (37.9%)	304 (13.6%)	3.9 (3.1-4.8)	Diabetes	1966 (49.7%)	7456 (37.7%)	1.6 (1.5-1.7)
	Diabetes	145 (32.5%)	443 (19.9%)	1.9 (1.6-2.4)	GERD/Peptic ulcer	1726 (43.6%)	4625 (23.4%)	2.5 (2.4-2.7)
	Hyperlipidemia	85 (19.1%)	429 (19.2%)	1.0 (0.8-1.3)	Hyperlipidemia	1341 (33.9%)	7918 (40.0%)	0.8 (0.7-0.8)
	HCV	82 (18.4%)	19 (0.8%)	26.2 (15.7-43.7)	HCV	1235 (31.2%)	369 (1.9%)	23.9 (21.1-27.0)
	Anemia	54 (12.1%)	159 (7.1%)	1.8 (1.3-2.5)	Alcohol Dependence	1004 (25.4%)	544 (2.8%)	12.0 (10.8-13.4)
	Alcohol Dependence	50 (11.2%)	31 (1.4%)	9.0 (5.7-14.2)	Steatosis of liver	550 (13.9%)	349 (1.8%)	9.0 (7.8-10.3)
	Anxiety	49 (11.0%)	146 (6.5%)	1.8 (1.3-2.5)	Hypothyroidism	495 (12.5%)	1582 (8.0%)	1.6 (1.5-1.8)
	Steatosis of liver	48 (10.8%)	41 (1.8%)	6.4 (4.2-9.9)	Asthma	411 (10.4%)	1908 (9.6%)	1.1 (1.0-1.2)
White	Hypertension	202 (43.4%)	358 (15.4%)	4.2 (3.4-5.2)	Hypertension	3791 (66.8%)	11,705 (41.3%)	2.9 (2.7-3.0)
	GERD/Peptic ulcer	161 (34.5%)	240 (10.3%)	4.6 (3.6-5.8)	Cirrhosis	2187 (38.6%)	230 (0.8%)	76.8 (66.7-88.3)
	Cirrhosis	128 (27.5%)	4 (0.2%)	220.2 (80.9-599.6)	GERD/Peptic ulcer	1970 (34.7%)	5750 (20.3%)	2.1 (2.0-2.2)
	Hyperlipidemia	89 (19.1%)	325 (13.9%)	1.5 (1.1-1.9)	Hyperlipidemia	1900 (33.5%)	10,550 (37.2%)	0.9 (0.8-0.9)
	Diabetes	87 (18.7%)	187 (8.0%)	2.6 (2.0-3.5)	Diabetes	1870 (33.0%)	5933 (20.9%)	1.9 (1.7-2.0)
	Anxiety	63 (13.5%)	193 (8.3%)	1.7 (1.3-2.3)	HCV	1780 (31.4%)	351 (1.2%)	36.5 (32.4-41.1)
	Asthma	58 (12.4%)	189 (8.1%)	1.6 (1.2-2.2)	Hypothyroidism	837 (14.8%)	2990 (10.5%)	1.5 (1.4-1.6)
	Hypothyroidism	43 (9.2%)	138 (5.9%)	1.6 (1.1-2.3)	Alcohol Dependence	829 (14.6%)	603 (2.1%)	7.9 (7.1-8.8)
	HCV	41 (8.8%)	14 (0.6%)	16.0 (8.6-29.5)	Asthma	667 (11.8%)	3114 (11.0%)	1.1 (1.0-1.2)
	Cholangitis	38 (8.2%)	3 (0.1%)	68.9 (21.2-224.1)	Osteoarthritis	427 (7.5%)	3415 (12.0%)	0.6 (0.5-0.7)
Other/Unknown	Hypertension	111 (35.5%)	242 (15.5%)	3.0 (2.3-3.9)	Hypertension	2392 (63.6%)	7345 (39.0%)	2.7 (2.5-2.9)
	GERD/Peptic ulcer	91 (29.1%)	120 (7.7%)	4.9 (3.6-6.7)	Cirrhosis	1478 (39.3%)	149 (0.8%)	81.0 (68.1-96.4)
	Cirrhosis	84 (26.8%)	3 (0.2%)	191.0 (59.9-609.2)	Diabetes	1334 (35.4%)	4613 (24.5%)	1.7 (1.6-1.8)
	Diabetes	61 (19.5%)	188 (12.0%)	1.8 (1.3-2.4)	GERD/Peptic ulcer	1286 (34.2%)	3301 (17.5%)	2.4 (2.3-2.6)
	Hyperlipidemia	54 (17.2%)	229 (14.6%)	1.2 (0.9-1.7)	HCV	1174 (31.2%)	346 (1.8%)	24.2 (21.3-27.5)
	HBV	38 (12.1%)	4 (0.3%)	53.9 (19.1-152.3)	Hyperlipidemia	1170 (31.1%)	6152 (32.7%)	0.9 (0.9-1.0)
	HCV	33 (10.5%)	6 (0.4%)	30.6 (12.7-73.8)	Alcohol Dependence	507 (13.5%)	312 (1.7%)	9.2 (8.0-10.7)
	Anxiety	21 (6.7%)	98 (6.3%)	1.1 (0.7-1.8)	Asthma	378 (10.0%)	1680 (8.9%)	1.1 (1.0-1.3)
	Asthma	21 (6.7%)	107 (6.8%)	1.0 (0.6-1.6)	Hypothyroidism	368 (9.8%)	1356 (7.2%)	1.4 (1.2-1.6)
	Vitamin D Deficiency	21 (6.7%)	55 (3.5%)	2.0 (1.2-3.3)	Steatosis of liver	237 (6.3%)	118 (0.6%)	10.6 (8.5-13.3)

*Note:* OR, odds ratio; HBV, Hepatitis B Virus; HCV, Hepatitis C Virus; GERD, Gastroesophageal Reflux Disease.

^a^No controls with HCV infection in the API group.

The predictive performance of ML models for EOLC varied across racial groups, as illustrated in Table 3. Models trained for API (AUC = 0.90, F1 = 0.77) and Hispanic (AUC = 0.92, F1 = 0.77) patients showed higher performance compared to those for White patients (AUC = 0.87, F1 = 0.64), despite the larger sample size in the White group. Models for early-onset HCC performed better than the general early-onset liver cancer models. Similarly, the model achieved higher AUC and F1 scores for API (AUC = 0.86, F1 = 0.81) and Hispanic (AUC = 0.92, F1 = 0.84) patients, with lower performance for White patients (AUC = 0.82, F1 = 0.64).

**Table 3.** Early-Onset Liver Cancer and HCC Prediction Model Performance

Metrics		All	Asian and pacific islander	Hispanic	White	Other/Unknown
Early-onset liver cancer model with the highest F1 score^a^		Logistic regression	Logistic regression	XGBoost	Logistic regression	Logistic regression
Validation dataset	AUC	0.85 (0.82, 0.88)	0.90 (0.85, 0.94)	0.92 (0.89, 0.95)	0.87 (0.82, 0.91)	0.81 (0.74, 0.88)
	F1 score	0.67 (0.62, 0.72)	0.77 (0.68, 0.85)	0.77 (0.69, 0.84)	0.64 (0.52, 0.71)	0.68 (0.58, 0.77)
Test dataset	AUC	0.86 (0.84, 0.89)	0.78 (0.71, 0.85)	0.81 (0.75, 0.87)	0.78 (0.71, 0.84)	0.79 (0.72, 0.86)
	F1 score	0.66 (0.61, 0.70)	0.65 (0.55, 0.74)	0.62 (0.51, 0.71)	0.53 (0.42, 0.64)	0.58 (0.42, 0.70)
Early-onset HCC model with the highest F1 score^a^		Logistic regression	Logistic regression	XGBoost	XGBoost	Logistic regression
Validation dataset	AUC	0.88 (0.85, 0.91)	0.86 (0.77, 0.93)	0.92 (0.87, 0.96)	0.82 (0.74, 0.89)	0.94 (0.88, 0.98)
	F1 score	0.70 (0.64, 0.75)	0.81 (0.72, 0.89)	0.84 (0.76, 0.91)	0.64 (0.51, 0.76)	0.81 (0.70, 0.90)
Test dataset	AUC	0.90 (0.87, 0.93)	0.89 (0.83, 0.94)	0.89 (0.82, 0.94)	0.76 (0.66, 0.85)	0.80 (0.69, 0.89)
	F1 score	0.76 (0.70, 0.81)	0.71 (0.60, 0.81)	0.76 (0.65, 0.85)	0.51 (0.33, 0.66)	0.60 (0.41, 0.75)

*Note:* HCC, Hepatocellular carcinoma; AUC, Area under the curve.

^a^Classification methods, including decision trees, random forests, logistic regression, XGBoost, and LightGBM, were employed. The method with the highest F1 score on the validation dataset was selected for final evaluation and reporting.

**Figure 1.**
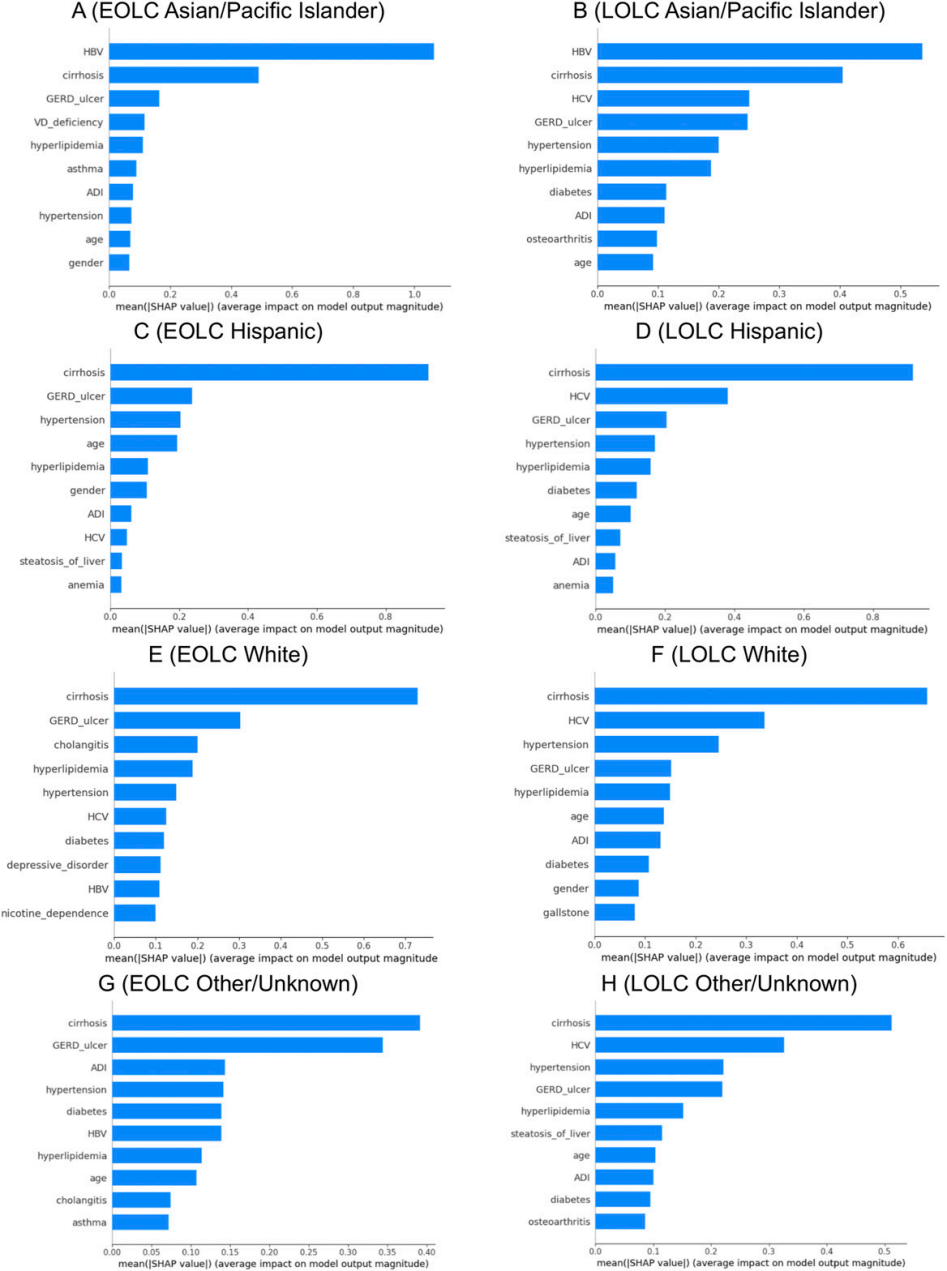
Race/ethnicity-specific feature importance plots based on SHAP values. Panels A and B represent the EOLC and LOLC models for Asian/Pacific Islanders, Panels C and D for Hispanics, Panels E and F for Whites, and Panels G and H for Other/Unknown. *Note*. EOLC, early-onset liver cancer; LOLC, late-onset liver cancer; HBV, Hepatitis B Virus; GERD, Gastroesophageal Reflux Disease; VD, Vitamin D; ADI, Area Deprivation Index; HCV, Hepatitis C Virus; SHAP, SHapley Additive exPlanations

The feature importance plots based on SHAP values reveal the most important comorbidities by race/ethnicity associated with EOLC and LOLC (Figure 1). For API patients, HBV and cirrhosis showed the highest mean SHAP values, with HBV reaching up to 1.0 in younger populations. However, in older populations, the mean SHAP value for HBV was around 0.5, and the importance of other comorbidities—such as cirrhosis, GERD/ulcer, hypertension, and hyperlipidemia—became more pronounced. Vitamin D deficiency and asthma also appear among the important factors in the EOLC, although they have much lower mean SHAP values compared to HBV and cirrhosis. In Hispanic patients, cirrhosis, GERD/ulcer, hypertension, hyperlipidemia, and HCV showed high mean SHAP values in both early and later stages, suggesting that HCV infection and metabolic syndrome-related risks are more prominent in this population. White patients exhibit a more discrete importance of comorbidity pattern, with generally lower mean SHAP values across comorbidities. Apart from cirrhosis, metabolic disease, HCV infection, cholangitis, mental health disorders, and nicotine dependence were also important predictors in young White liver cancer patients. The relatively lower and more evenly spread SHAP values across conditions in White patients suggest a more heterogeneous comorbidity profile, consistent with the model’s lower F1 and AUC score for this group. Race/ethnicity-specific feature importance plots associated with early-onset HCC are displayed in Figure S2.

## Discussion

EOLC patients exhibited distinct comorbidity profiles by race/ethnicity groups, with HBV infection as the predominant comorbidity in API patients, HCV infection and metabolic disorder-related comorbidities playing significant roles in Hispanic patients, and a more diverse, less concentrated comorbidity profile in White patients. To our knowledge, this is the first study to comprehensively evaluate the comorbidity patterns of EOLC patients by race/ethnicity, providing further insights into the etiology of EOLC and supporting targeted strategies for liver cancer prevention in young populations.

Racial and ethnic disparities in hepatitis virus infections contribute to varying levels of liver cancer risk among different racial/ethnic groups. HBV infection is the most significant comorbidity among API patients. Although chronic HBV infection rates are generally low in the U.S. (<1%), the increased immigration from HBV-endemic regions, such as East Asia and the Pacific Islands, might have led to the rising prevalence of HBV in this population.^34,35^ Immigrants from these areas face chronic HBV risks similar to those in their home countries, where hepatitis B surface antigen prevalence exceeds 2%.^36-39^ Screening and vaccination for HBV in this community is also inadequate. A survey of Asian American primary care providers revealed that 50% did not routinely screen all their Asian patients for HBV. Additionally, over 80% of these providers reported that less than half of their adult Asian patients had received the HBV vaccine.^40^ In addition, the stigma of HBV infection can further prevent efforts to improve vaccination coverage and early screening, exacerbating the risk of chronic infection and liver cancer.^41^ Etiologically, HBV can integrate near oncogenes, altering gene expression or function and promoting malignant transformation without cirrhosis, which may contribute to the early onset of liver cancer.^42^ In contrast, HCV infection is the most prevalent in Hispanics, with a prevalence of 1.5%, which might be associated with higher rates of illicit drug use and limited access to testing and treatment services in this group.^43,44^ Additionally, socioeconomic disadvantages—such as lower income levels and reduced access to healthcare—impede early diagnosis and treatment of HCV, further contributing to elevated infection rates.^45^ We also observed that HCV prevalence is relatively lower in EOLC cases than in LOLC cases. This finding aligns with the National Health and Nutrition Examination Survey (NHANES) data, which indicates that individuals aged 55-64 are 6.4 times more likely to have active HCV infection than those aged 18-40.^46^ The lower rate of spontaneous viral clearance among older adults may partly explain this discrepancy.^47^ Furthermore, as blood screening for HCV began in 1990, many older individuals may have acquired the virus through medical procedures or intravenous drug use before the implementation of widespread preventive measures.^48^

Apart from hepatitis virus infections, non-infectious comorbidities also display distinct racial disparities. Metabolic conditions, such as hypertension and diabetes, are most prevalent among Hispanics, which results from a combination of genetic, lifestyle, and socioeconomic factors.^49^ For instance, the R230 C variant in Hispanic individuals has been linked to low High-Density Lipoprotein Cholesterol (HDL-C) levels, while a rare Adiponectin, C1Q And Collagen Domain Containing (ADIPOQ) gene mutation is associated with increased risks of heart disease and insulin resistance.^50^ These factors may contribute to the high prevalence of metabolic disorders and the high obesity rate of 43.7-47% among Hispanic adults.^51-53^ Socioeconomic barriers further exacerbate the status of metabolic disorders, as many Hispanic individuals face challenges such as lack of health insurance, limited English proficiency, and low education or literacy levels.^51,54^ In addition, communities with lower socioeconomic status often experience reduced access to nutritious food and safe living environments, increasing the risk of developing chronic metabolic diseases.^55^ In contrast, mental disorders are more prominent in Whites, particularly among younger individuals. This pattern aligns with findings from the general population, where lifetime prevalence rates of mental disorders were highest among Whites (45.6%), followed by Latinos (38.8%) and Blacks (37.0%).^56-60^ Additionally, cultural differences and stigma can affect how mental disorders are reported, potentially leading to lower rates in the racial minorities but higher rates in Whites.^56,61,62^ Moreover, asthma and hypothyroidism are more common among Whites, consistent with their higher prevalence rates of 9.4% and 8.1%, respectively, in this population.^63,64^ Cholangitis, particularly primary sclerosing cholangitis (PSC)—a significant risk factor for hepatobiliary cancer—also shows a higher prevalence among younger White patients.^65-67^ Notably, the incidence of PSC has been rising in several countries, which may contribute to the increasing trend of EOLC.^68-70^ Intriguingly, many of those comorbidities involve autoimmune and inflammatory pathological processes, with their heightened prevalence likely influenced by a combination of genetic susceptibility, socioeconomic factors, and environmental influences. For example, the HLA-Cw*0701 allele is associated with genetic susceptibility to primary sclerosing cholangitis in Whites.^71^ Furthermore, previous studies have identified that higher socioeconomic status and education levels of White individuals are independently associated with increased risks of thyroid disease.^64^ Collectively, these results underscore the etiological heterogeneity of EOLC and support the need for risk assessment and clinical surveillance strategies that reflect the predominant comorbidity patterns within each racial group. Understanding that liver cancer may develop through different pathways—such as viral, metabolic, autoimmune, or psychosocial—depending on the population context is essential for informing precision prevention efforts.

The use of machine learning and SHAP values enabled us to quantify racial disparities in comorbidity risk profiles. Our findings showed that race- and ethnicity-specific models for API and Hispanic patients outperformed those for White patients. Additionally, the HCC model demonstrated superior performance compared to the general liver cancer models, highlighting the importance of tailored ML approaches that account for specific racial groups and cancer subtypes. Such models may enable more accurate risk assessments and provide insights for targeted prevention efforts. For example, despite the global implementation of universal HBV vaccination since 2008, HBV remains a dominant risk factor for liver cancer among API patients, particularly in younger populations.^72^ This highlights the need for enhancing vaccination coverage and early screening in API communities, as well as continued efforts to identify and treat chronic HBV infections. Validating HBV screening results and ensuring timely follow-up care is also crucial for effectively managing chronic HBV infections and reducing the risk of liver cancer in these populations.^73^ For Hispanic communities, targeted interventions aimed at increasing access to HCV screening and treatment, along with preventing metabolic diseases and obesity, are essential.^74^ Notably, culturally sensitive interventions have been shown to improve the metabolic health of Hispanic participants, as evidenced by reductions in body mass index (BMI), blood pressure, lipid levels, and hemoglobin A1c.^75^ In contrast, the more diverse comorbidity patterns observed in White patients may reflect broader genetic, socioeconomic, and lifestyle diversity, which contributes to a range of conditions impacting early diagnosis. These findings suggest a need for public health interventions that address a broader spectrum of risk factors, extending beyond hepatitis virus infections and metabolic disorders. In particular, psychosocial and immune-mediated risk factors warrant further exploration and targeted prevention efforts.

The performance metrics, including F1 scores and AUC values, highlighted the effectiveness of our machine learning models in predicting EOLC risk across diverse racial and ethnic groups. High F1 scores reflect a balance between precision and sensitivity, while robust AUC values underscore the models’ discriminative power. These findings are consistent with the results from the existing liver cancer prediction studies. For example, a machine learning model combining soft ensembles of random forest, XGBoost, and logistic regression achieved an AUC of 0.872 for predicting HCC risk in chronic hepatitis B patients on antiviral therapy.^76^ Another study, using data from 377,065 participants in the NIH-AARP Diet and Health Study, applied a RUSBoosted Trees model and reported an AUC of 0.72 in the training sample and 0.65 in the validation sample for HCC risk prediction.^77^ While differences in datasets and study designs limit direct comparisons, our models showed competitive performance and underscored the value of incorporating racial and ethnic factors to develop more equitable, population-specific prediction strategies.

There are several limitations to this study. The UCHDW dataset is not population-based and only captures patient care data generated at the UC Health system. This limits our ability to access the complete medical history of all included patients. To address this limitation, we employed a comprehensive strategy to identify comorbidities, including diagnosis codes, lab test results, and history of specific medications. We also included only those patients who stayed relatively persistent within the UC Health system, defined as having at least two visits recorded in the dataset, to ensure sufficient periods of exposure. Furthermore, the missing rates for behavioral variables (e.g., smoking, alcohol consumption) and BMI were high among young patients, preventing us from assessing the impact of those factors on EOLC prediction. To mitigate this, we included diagnoses of comorbidities such as alcohol and nicotine dependence, as well as metabolic syndrome-related comorbidities, as proxy variables. Additionally, there is potential for bias related to SES and access to healthcare services. Patients with limited access or inadequate insurance coverage may be underrepresented, which could affect the observed prevalence and detection of comorbidities. Although we included the ADI as a proxy for neighborhood-level SES, residual confounding may persist. This limitation should be considered when interpreting the generalizability of our findings.

## Conclusions

Collectively, our study underscores the disparity in EOLC risk profiles across racial and ethnic groups and the value of ML in identifying these complex patterns. The results show that HBV infection is the primary comorbidity among API patients, and Hispanic patients are notably affected by HCV and metabolic disorders. In addition, White patients exhibit a broader, less concentrated comorbidity pattern, with mental health disorders and inflammatory conditions also playing important roles. Targeted strategies for those comorbidities are needed to prevent liver cancer in young populations.

## Supplemental Material

Supplemental Material - Racial Disparities in Comorbidity Patterns of Early-Onset Liver Cancer: A Machine Learning Analysis Supplemental Material for Racial Disparities in Comorbidity Patterns of Early-Onset Liver Cancer: A Machine Learning Analysis by Bingya Ma, Kai Zheng, Fa-Chyi Lee, Yunxia Lu in Cancer Control.

## Data Availability

The data used in this study contain de-identified patient information and are not publicly available.*

## References

[1] Bray F Laversanne M Sung H , et al. Global cancer statistics 2022: GLOBOCAN estimates of incidence and mortality worldwide for 36 cancers in 185 countries. CA Cancer J Clin. 2024;74(3):229-263. doi:10.3322/caac.21834 38572751

[2] Makarova‐Rusher OV Altekruse SF McNeel TS , et al. Population attributable fractions of risk factors for hepatocellular carcinoma in the United States. Cancer. 2016;122(11):1757-1765. doi:10.1002/cncr.29971 26998818 PMC5548177

[3] Ashktorab H Kupfer SS Brim H Carethers JM . Racial disparity in gastrointestinal cancer risk. Gastroenterology. 2017;153(4):910-923. doi:10.1053/j.gastro.2017.08.018 28807841 PMC5623134

[4] Siegel RL Giaquinto AN Jemal A . Cancer statistics, 2024. CA Cancer J Clin. 2024;74(1):12-49. doi:10.3322/caac.21820 38230766

[5] Siegel RL Kratzer TB Giaquinto AN Sung H Jemal A . Cancer statistics. CA Cancer J Clin. 2025;75(1):10-45. doi:10.3322/caac.21871 39817679 PMC11745215

[6] Hsieh MC Ratnapradipa KL Rozek L Wen S Chiu YW Peters ES . Temporal trends and patterns for early- and late-onset adult liver cancer incidence vary by race/ethnicity, subsite, and histologic type in the United States from 2000 to 2019. Cancer Causes Control. 2025;36(5):551-560. doi:10.1007/s10552-024-01955-4 39786651 PMC11982089

[7] Koh B Tan DJH Ng CH , et al. Patterns in cancer incidence among people younger than 50 Years in the US, 2010 to 2019. JAMA Netw Open. 2023;6(8):e2328171. doi:10.1001/jamanetworkopen.2023.28171 37585204 PMC10433086

[8] Ugai T Sasamoto N Lee HY , et al. Is early-onset cancer an emerging global epidemic? Current evidence and future implications. Nat Rev Clin Oncol. 2022;19(10):656-673. doi:10.1038/s41571-022-00672-8 36068272 PMC9509459

[9] Chang PE Ong WC Lui HF Tan CK . Is the prognosis of young patients with hepatocellular carcinoma poorer than the prognosis of older patients? A comparative analysis of clinical characteristics, prognostic features, and survival outcome. J Gastroenterol. 2008;43(11):881-888. doi:10.1007/s00535-008-2238-x 19012042

[10] Park CH Jeong SH Yim HW , et al. Family history influences the early onset of hepatocellular carcinoma. World J Gastroenterol. 2012;18(21):2661-2667. doi:10.3748/wjg.v18.i21.2661 22690075 PMC3370003

[11] Wang Q Luan W Villanueva GA , et al. Clinical prognostic variables in young patients (under 40 years) with hepatitis B virus‐associated hepatocellular carcinoma. J Dig Dis. 2012;13(4):214-218. doi:10.1111/j.1751-2980.2012.00577.x 22435506

[12] Wan DW Tzimas D Smith JA , et al. Risk factors for early-onset and late-onset hepatocellular carcinoma in asian Immigrants with hepatitis B in the United States. Am J Gastroenterol. 2011;106(11):1994-2000. doi:10.1038/ajg.2011.302 21912436

[13] Goh MJ Kang W Kim KM , et al. Incidence and risk factors for development of hepatocellular carcinoma at young age in patients with chronic hepatitis B. Scand J Gastroenterol. 2022;57(1):70-77. doi:10.1080/00365521.2021.1988700 34731072

[14] Nakano M Kawaguchi T Nakamoto S , et al. Effect of occult hepatitis B virus infection on the early-onset of hepatocellular carcinoma in patients with hepatitis C virus infection. Oncol Rep. 2013;30(5):2049-2055. doi:10.3892/or.2013.2700 23982634

[15] Bennett M Kleczyk E J Hayes K Mehta R . Evaluating similarities and differences between machine learning and traditional statistical modeling in healthcare analytics. In: Antonio Aceves Fernandez MM Travieso-Gonzalez C , (eds.), Artificial Intelligence. IntechOpen. 2022. doi:10.5772/intechopen.105116

[16] Bhatia HS Hurst S Desai P Zhu W Yeang C . Lipoprotein(a) testing trends in a large academic health system in the United States. J Am Heart Assoc. 2023;12(18):e031255. doi:10.1161/JAHA.123.031255 37702041 PMC10547299

[17] Kim Y Tian Y Yang J , et al. Comparative safety and effectiveness of alendronate versus raloxifene in women with osteoporosis. Sci Rep. 2020;10(1):11115. doi:10.1038/s41598-020-68037-8 32632237 PMC7338498

[18] Rasmussen-Torvik LJ Furmanchuk A Stoddard AJ , et al. The effect of number of healthcare visits on study sample selection in electronic health record data. Int J Popul Data Sci. 2020;5(1):1156. doi:10.23889/ijpds.v5i1.1156 32864475 PMC7448749

[19] Hennessy S Bilker WB Berlin JA Strom BL . Factors influencing the optimal control-to-case ratio in matched case-control studies. Am J Epidemiol. 1999;149(2):195-197. doi:10.1093/oxfordjournals.aje.a009786 9921965

[20] von Elm E Altman DG Egger M , et al. The Strengthening the Reporting of Observational Studies in Epidemiology (STROBE) statement: guidelines for reporting observational studies. Ann Intern Med. 2007;147(8):573-577. doi:10.7326/0003-4819-147-8-200710160-00010 17938396

[21] Kind AJH Buckingham WR . Making neighborhood-disadvantage metrics accessible - the neighborhood atlas. N Engl J Med. 2018;378(26):2456-2458. doi:10.1056/NEJMp1802313 29949490 PMC6051533

[22] Liu R Durbin-Johnson B Paciotti B , et al. Metabolic dysfunctions predict the development of Alzheimer’s disease: statistical and machine learning analysis of EMR data. Alzheimer's Dement. 2024;20(10):6765-6775. doi:10.1002/alz.14101 39140368 PMC11485292

[23] Stekhoven DJ Bühlmann P . MissForest--non-parametric missing value imputation for mixed-type data. Bioinforma Oxf Engl. 2012;28(1):112-118. doi:10.1093/bioinformatics/btr597 22039212

[24] Ording AG Sørensen HT . Concepts of comorbidities, multiple morbidities, complications, and their clinical epidemiologic analogs. Clin Epidemiol. 2013;5:199-203. doi:10.2147/CLEP.S45305 23861599 PMC3704301

[25] Maresca R Mignini I Varca S , et al. Inflammatory bowel diseases and non-alcoholic fatty liver disease: piecing a complex puzzle together. Int J Mol Sci. 2024;25(6):3278. doi:10.3390/ijms25063278 38542249 PMC10970310

[26] van Munster KN Bergquist A Ponsioen CY . Inflammatory bowel disease and primary sclerosing cholangitis: one disease or two? J Hepatol. 2024;80(1):155-168. doi:10.1016/j.jhep.2023.09.031 37940453

[27] Zessner-Spitzenberg J Ferlitsch A Waldmann E , et al. Detection of high-risk polyps at screening colonoscopy indicates risk for liver and biliary cancer death. Dig Liver Dis. 2024;56(3):502-508. doi:10.1016/j.dld.2023.08.051 37704511

[28] Noh B Park YM Kwon Y , et al. Machine learning-based survival rate prediction of Korean hepatocellular carcinoma patients using multi-center data. BMC Gastroenterol. 2022;22(1):85. doi:10.1186/s12876-022-02182-4 35220946 PMC8882306

[29] Zuo D Yang L Jin Y Qi H Liu Y Ren L . Machine learning-based models for the prediction of breast cancer recurrence risk. BMC Med Inf Decis Making. 2023;23(1):276. doi:10.1186/s12911-023-02377-z PMC10688055 38031071

[30] Databricks . What is AutoML? | Databricks documentation. 2025. https://docs.databricks.com/aws/en/machine-learning/automl (Accessed April 21, 2025).

[31] Riyanto S Sitanggang IS Djatna T Atikah TD . Comparative analysis using various performance metrics in imbalanced data for multi-class text classification. Int J Adv Comput Sci Appl. 2023;14(6):01406116. doi:10.14569/IJACSA.2023.01406116

[32] Fryer DV Strumke I Nguyen H . Model independent feature attributions: Shapley values that uncover non-linear dependencies. PeerJ Comput Sci. 2021;7:e582. doi:10.7717/peerj-cs.582 PMC8189022 34151001

[33] Cho Y Cho EJ Yoo JJ , et al. Association between lipid profiles and the incidence of hepatocellular carcinoma: a nationwide population-based study. Cancers. 2021;13(7):1599. doi:10.3390/cancers13071599 33808412 PMC8037932

[34] Kowdley KV Wang CC Welch S Roberts H Brosgart CL . Prevalence of chronic hepatitis B among foreign-born persons living in the United States by country of origin. Hepatol Baltim Md. 2012;56(2):422-433. doi:10.1002/hep.24804 22105832

[35] Razavi-Shearer D Gamkrelidze I Pan CQ , et al. The impact of immigration on hepatitis B burden in the United States: a modelling study. Lancet Reg Health Am. 2023;22:100516. doi:10.1016/j.lana.2023.100516 37274551 PMC10239007

[36] Mitchell T Armstrong GL Hu DJ Wasley A Painter JA . The increasing burden of imported chronic hepatitis B--United States, 1974-2008. PLoS One. 2011;6(12):e27717. doi:10.1371/journal.pone.0027717 22163270 PMC3233539

[37] Weinbaum CM Williams I Mast EE , et al. Recommendations for identification and public health management of persons with chronic hepatitis B virus infection. MMWR Recomm Rep (Morb Mortal Wkly Rep). 2008;57(RR-8):1-20. 18802412

[38] Ward JW Byrd KK . Hepatitis B in the United States: a major health disparity affecting many foreign-born populations. Hepatol Baltim Md. 2012;56(2):419-421. doi:10.1002/hep.25799 22532028

[39] Sarkar M Shvachko VA Ready JB , et al. Characteristics and management of patients with chronic hepatitis B in an integrated care setting. Dig Dis Sci. 2014;59(9):2100-2108. doi:10.1007/s10620-014-3142-2 24728968 PMC4149592

[40] Chu D Yang JD Lok AS , et al. Hepatitis B screening and vaccination practices in asian american primary care. Gut Liver. 2013;7(4):450-457. doi:10.5009/gnl.2013.7.4.450 23898386 PMC3724034

[41] Freeland C Wallace J Wang S , et al. The urgent need to end hepatitis B stigma and discrimination. Lancet Gastroenterol Hepatol. 2025;10(2):105-107. doi:10.1016/S2468-1253(24)00389-3 39706211

[42] Neuveut C Wei Y Buendia MA . Mechanisms of HBV-related hepatocarcinogenesis. J Hepatol. 2010;52(4):594-604. doi:10.1016/j.jhep.2009.10.033 20185200

[43] Cummins CA Erlyana E Fisher DG Reynolds GL . Hepatitis C infection among Hispanics in California. J Addict Dis. 2015;34(4):263-273. doi:10.1080/10550887.2015.1074500 26372008 PMC4727747

[44] Kuniholm MH Jung M Everhart JE , et al. Prevalence of hepatitis C virus infection in US Hispanic/Latino adults: results from the NHANES 2007-2010 and HCHS/SOL studies. J Infect Dis. 2014;209(10):1585-1590. doi:10.1093/infdis/jit672 24423693 PMC3997577

[45] Velasco-Mondragon E Jimenez A Palladino-Davis AG Davis D Escamilla-Cejudo JA . Hispanic health in the USA: a scoping review of the literature. Public Health Rev. 2016;37:31. doi:10.1186/s40985-016-0043-2 29450072 PMC5809877

[46] Lewis KC Barker LK Jiles RB Gupta N . Estimated prevalence and awareness of hepatitis C virus infection among US adults: national health and nutrition examination survey, january 2017-march 2020. Clin Infect Dis. 2023;77(10):1413-1415. doi:10.1093/cid/ciad411 37417196 PMC11000503

[47] Aisyah DN Shallcross L Hully AJ O’Brien A Hayward A . Assessing hepatitis C spontaneous clearance and understanding associated factors-A systematic review and meta-analysis. J Viral Hepat. 2018;25(6):680-698. doi:10.1111/jvh.12866 29345844

[48] Hsu HH Gonzalez M Foung SK Feinstone SM Greenberg HB . Antibodies to hepatitis C virus in low-risk blood donors: implications for counseling positive donors. Gastroenterology. 1991;101(6):1724-1727. doi:10.1016/0016-5085(91)90413-f 1720106

[49] Akam EY Nuako AA Daniel AK Stanford FC . Racial disparities and cardiometabolic risk: new horizons of intervention and prevention. Curr Diabetes Rep. 2022;22(3):129-136. doi:10.1007/s11892-022-01451-6 PMC9908372 35175453

[50] Fernandez ML . Lifestyle factors and genetic variants associated to health disparities in the hispanic population. Nutrients. 2021;13(7):2189. doi:10.3390/nu13072189 34202120 PMC8308310

[51] Zhang Y Chen GC Sotres-Alvarez D , et al. General or central obesity and mortality among US hispanic and latino adults. JAMA Netw Open. 2024;7(1):e2351070. doi:10.1001/jamanetworkopen.2023.51070 38227314 PMC10792478

[52] Howell CR Juarez L Agne AA , et al. Assessing hispanic/latino and non-hispanic white social determinants of obesity among a community sample of residents in the rural southeast US. J Immigr Minority Health. 2022;24(6):1469-1479. doi:10.1007/s10903-022-01334-8 PMC9980419 35174428

[53] Hales CM . Prevalence of obesity and severe obesity among adults: United States, 2017–2018. NCHS Data Brief. 2020;360:1-8. 32487284

[54] Davidson JA Moreno PR Badimon JJ , et al. Cardiovascular disease prevention and care in Latino and Hispanic subjects. Endocr Pract. 2007;13(1):77-85. doi:10.4158/EP.13.1.77 17360307

[55] Odoms-Young A Brown AGM Agurs-Collins T Glanz K . Food insecurity, neighborhood food environment, and health disparities: state of the science, research gaps and opportunities. Am J Clin Nutr. 2024;119(3):850-861. doi:10.1016/j.ajcnut.2023.12.019 38160801 PMC10972712

[56] Asnaani A Richey JA Dimaite R Hinton DE Hofmann SG . A cross-ethnic comparison of lifetime prevalence rates of anxiety disorders. J Nerv Ment Dis. 2010;198(8):551-555. doi:10.1097/NMD.0b013e3181ea169f 20699719 PMC2931265

[57] Goodwin RD Weinberger AH Kim JH Wu M Galea S . Trends in anxiety among adults in the United States, 2008-2018: rapid increases among young adults. J Psychiatr Res. 2020;130:441-446. doi:10.1016/j.jpsychires.2020.08.014 32905958 PMC7441973

[58] Assari S . Race, sense of control over life, and short-term risk of mortality among older adults in the United States. Arch Med Sci. 2017;13(5):1233-1240. doi:10.5114/aoms.2016.59740 28883866 PMC5575207

[59] Assari S Moazen-Zadeh E Lankarani MM Micol-Foster V . Race, depressive symptoms, and all-cause mortality in the United States. Front Public Health. 2016;4:40. doi:10.3389/fpubh.2016.00040 27014677 PMC4794497

[60] Alvarez K Fillbrunn M Green JG , et al. Race/ethnicity, nativity, and lifetime risk of mental disorders in US adults. Soc Psychiatr Psychiatr Epidemiol. 2019;54(5):553-565. doi:10.1007/s00127-018-1644-5 PMC6586416 30547212

[61] Johnson MR Hartzema AG Mills TL , et al. Ethnic differences in the reliability and validity of a panic disorder screen. Ethn Health. 2007;12(3):283-296. doi:10.1080/13557850701235069 17454101

[62] Eylem O de Wit L van Straten A , et al. Stigma for common mental disorders in racial minorities and majorities a systematic review and meta-analysis. BMC Public Health. 2020;20(1):879. doi:10.1186/s12889-020-08964-3 32513215 PMC7278062

[63] Swed S Sawaf B Al-Obeidat F , et al. Asthma prevalence among United States population insights from NHANES data analysis. Sci Rep. 2024;14(1):8059. doi:10.1038/s41598-024-58429-5 38580691 PMC10997649

[64] Zhang X Wang X Hu H Qu H Xu Y Li Q . Prevalence and trends of thyroid disease among adults, 1999-2018. Endocr Pract. 2023;29(11):875-880. doi:10.1016/j.eprac.2023.08.006 37619827

[65] Souza M Lima LCV Al-Sharif L Huang DQ . Incidence of hepatobiliary malignancies in primary sclerosing cholangitis: systematic review and meta-analysis. Clin Gastroenterol Hepatol. 2024;24:S1542-3565. doi:10.1016/j.cgh.2024.09.037 39709139

[66] Wang M Harris A McCulloch CE Wadhwani SI Lai JC Rubin JB . Racial differences in primary sclerosing cholangitis: a retrospective cohort study leveraging a new ICD-10 code. Ann Hepatol. 2025;30(1):101901. doi:10.1016/j.aohep.2025.101901 40081809 PMC12826390

[67] Rupp C Rössler A Zhou T , et al. Impact of age at diagnosis on disease progression in patients with primary sclerosing cholangitis. United Eur Gastroenterol J. 2018;6(2):255-262. doi:10.1177/2050640617717156 PMC5833225 29511555

[68] Folseraas T Boberg KM . Cancer risk and surveillance in primary sclerosing cholangitis. Clin Liver Dis. 2016;20(1):79-98. doi:10.1016/j.cld.2015.08.014 26593292

[69] Strömland K Nordin V Miller M Akerström B Gillberg C . Autism in thalidomide embryopathy: a population study. Dev Med Child Neurol. 1994;36(4):351-356. doi:10.1111/j.1469-8749.1994.tb11856.x 8157157

[70] Crothers H Ferguson J Quraishi MN Cooney R Iqbal TH Trivedi PJ . Past, current, and future trends in the prevalence of primary sclerosing cholangitis and inflammatory bowel disease across England (2015-2027): a nationwide, population-based study. Lancet Reg Health Eur. 2024;44:101002. doi:10.1016/j.lanepe.2024.101002 39099647 PMC11296053

[71] Moloney MM Thomson LJ Strettell MJ Williams R Donaldson PT . Human leukocyte antigen-C genes and susceptibility to primary sclerosing cholangitis. Hepatol Baltim Md. 1998;28(3):660-662. doi:10.1002/hep.510280309 9731555

[72] Ni YH . Natural history of hepatitis B virus infection: pediatric perspective. J Gastroenterol. 2011;46(1):1-8. doi:10.1007/s00535-010-0304-7 20812021

[73] Beckett GA Ramirez G Vanderhoff A , et al. Early identification and linkage to care of persons with chronic hepatitis B virus infection--three U.S. sites, 2012-2014. MMWR Morb Mortal Wkly Rep. 2014;63(18):399-401. 24807238 PMC5779400

[74] Wong A Le A Lee MH , et al. Higher risk of hepatocellular carcinoma in Hispanic patients with hepatitis C cirrhosis and metabolic risk factors. Sci Rep. 2018;8(1):7164. doi:10.1038/s41598-018-25533-2 29740031 PMC5940826

[75] Marks S . Culturally sensitive education can decrease hispanic workers’ risk of metabolic syndrome. Workplace Health Saf. 2016;64(11):543-549. doi:10.1177/2165079916634712 27059994

[76] Lee HW Kim H Park T , et al. A machine learning model for predicting hepatocellular carcinoma risk in patients with chronic hepatitis B. Liver Int. 2023;43(8):1813-1821. doi:10.1111/liv.15597 37452503

[77] Thomas J Liao LM Sinha R Patel T Antwi SO . Hepatocellular carcinoma risk prediction in the NIH-AARP Diet and health study cohort: a machine learning approach. J Hepatocell Carcinoma. 2022;9:69-81. doi:10.2147/JHC.S341045 35211426 PMC8858015

