# Supplementary material for: Racial Disparities in Comorbidity Patterns of Early-Onset Liver Cancer: A Machine Learning Analysis
Source: Cancer Control. 2025 Jul 30;32:10732748251363687. doi: 10.1177/10732748251363687 (PMC12317173; doi:10.1177/10732748251363687)
Supplement: Supplemental Material - Racial Disparities in Comorbidity Patterns of Early-Onset Liver Cancer: A Machine Learning Analysis [file sj-pdf-1-ccx-10.1177_10732748251363687.pdf]

Figure S1. Flow chart of the data collection process.

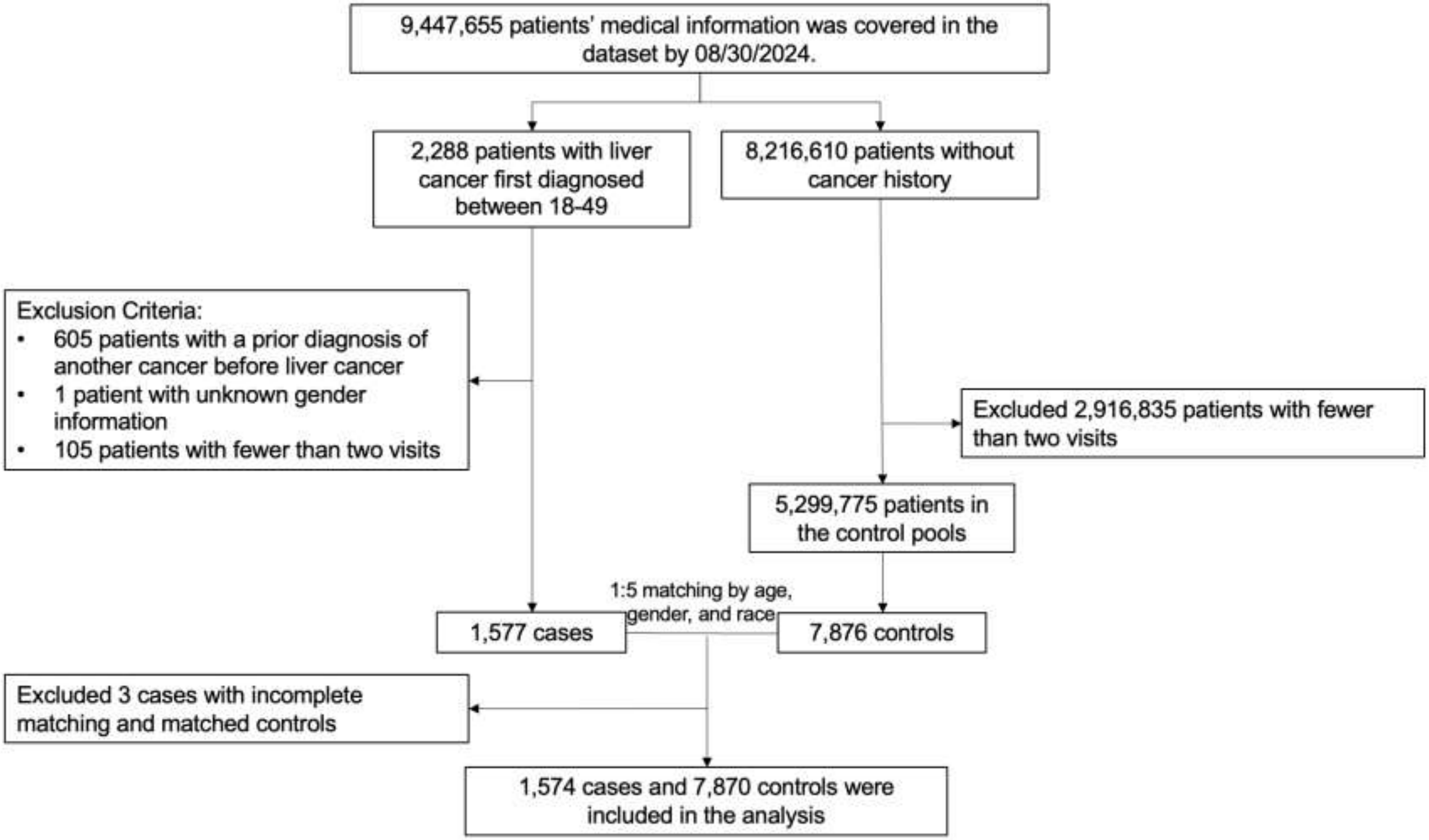

Table S1. Definition of Included Comorbidities

Comorbidity	Diagnosis	Lab tests (follow any of the criteria)	Medication
Hepatitis B Virus (HBV) Infection	✓	✓ Hepatitis B surface antigen (HBsAg) ≥ upper range or reactive/detected; Hepatitis B virus deoxyribonucleic acid (HBV DNA) ≥ upper range or reactive/detected	
Hepatitis D Virus (HDV) Infection	✓	✓ Hepatitis D virus antibody (HDV Ab) ≥ upper range, or reactive/detected; Hepatitis D virus ribonucleic acid (HDV RNA) ≥ upper range, or reactive/detected	
Hepatitis C Virus (HCV) Infection	✓	✓ Hepatitis C virus antibody (HCV Ab) ≥ upper range, or reactive/detected; Hepatitis C virus ribonucleic acid (HCV RNA) ≥ upper range, or reactive/detected	✓ Direct-acting antivirals (DAAs), including: Elbasvir/Grazoprevir, Glecaprevir/Pibrentasvir, Sofosbuvir/Ledipasvir, Velpatasvir/Voxilaprevir
Cirrhosis Of Liver	✓		
Steatosis Of Liver	✓		
Autoimmune Liver Disease	✓		
Prediabetes	✓	✓ Hemoglobin A1c (HbA1C) > upper range and ≤ 6.4%; fasting glucose > upper range and ≤ 125 mg/dL	
Diabetes	✓	✓ HbA1C > 6.4%; fasting glucose > 125 mg/dL; random glucose > 200 mg/dL	✓ Antidiabetic medication
Type 1 Diabetes	✓	✓ Insulin-related antibodies: Zinc transporter 8 antibody (ZnT8) ≥ upper range or reactive/detected; Glutamic acid decarboxylase antibody (GAB) ≥ upper range or reactive/detected; Islet cell antibody (ICA) ≥ upper range or reactive/detected; Insulin autoantibody (IAA) ≥ upper range or reactive/detected	✓ Glucagon

Type 2 Diabetes	✓		✓ Oral hypoglycemics
Hyperlipidemia	✓	✓ Triglycerides ≥ upper range; Total cholesterol ≥ upper range; Low-density lipoprotein (LDL) ≥ upper range; High-density lipoprotein (HDL) < lower range	✓ Lipid-modifying agents
Hypertension	✓	✓ Mean diastolic blood pressure ≥ 90 mmHg or systolic blood pressure ≥ 140 mmHg in three consecutive visit days	✓ Antihypertensive medications
Primary Hypertension	✓ Without diagnoses of secondary hypertension, and without diseases leading to secondary hypertension (kidney disease, obstructive sleep apnea, pheochromocytoma, primary aldosteronism, hypothyroidism, hyperparathyroidism, pituitary dependent hypercortisolism, secondary hypertension)		
Secondary Hypertension	✓ With diagnoses of secondary		

	hypertension, or with diseases leading to secondary hypertension		
Cholangitis	✓		
Primary Sclerosing Cholangitis	✓		
Gallstone	✓		
Cholesterolosis of Gallbladder	✓		
Anxiety	✓		
Depressive Disorder	✓		
GERD and Peptic Ulcer	✓		
Peptic Ulcer	✓		
Gastroesophageal Reflux Disease (GERD)	✓ Without peptic ulcer		
Proton Pump Inhibitors Users			✓ Excluding patients who had a diagnosis of cirrhosis or gastritis before PPI use to avoid PPI use related to these conditions.
Ulcerative Colitis	✓		
Crohn's Disease	✓		
Polyp Of Large Intestine	✓		
Diverticular Disease of The Colon	✓		
Chronic Kidney Disease	✓		
Kidney Stone	✓		
Prostatic Hyperplasia	✓		
Alcohol Dependence	✓		

Nicotine Dependence	✓		
Vitamin D Deficiency	✓	✓ 25-Hydroxyvitamin D3 + 25-Hydroxyvitamin D2 < lower range	
Hypothyroidism	✓	✓ Free thyroxine (fT4) ≤ lower range; Thyroid-stimulating hormone (TSH) ≥ upper range AND lower range < Free thyroxine (fT4) < upper range	✓ Levothyroxine
Human Immunodeficiency Virus (HIV) Infection	✓	✓ Human immunodeficiency virus antibody (HIV Ab) ≥ upper range or reactive/detected; Human immunodeficiency virus ribonucleic acid (HIV RNA) ≥ upper range or reactive/detected	
Cataract	✓		
Anemia	✓ Excluded diagnoses within one year of the index date due to the high likelihood of liver cancer-related anemia	✓ Hemoglobin < lower range (Excluded records within one year of the index date)	
Primary Anemia	✓ With nutritional anemia, hemolytic anemia, anemia due to decreased red cell production, anemia due to disturbance of hemoglobin synthesis (Excluded		

	diagnoses within one year of the index date)		
Chronic Diseases Related Anemia	✓ With disease of liver, disorder of gastrointestinal tract, chronic kidney disease, chronic infectious disease, autoimmune disease (Excluded diagnoses within one year of the index date)		
Cataract	✓		
Congenital Heart Disease	✓		
Cerebrovascular Disease	✓		
Myocardial Infarction	✓		
Peripheral Vascular Disease	✓		
Coronary Arteriosclerosis	✓		
Asthma	✓		✓ Using both beta-2 agonists and inhaled

			corticosteroids, excluding patients with COPD diagnosis
Obstructive Sleep Apnea (OSA)	✓		
Allergic Rhinitis	✓		
Chronic Obstructive Pulmonary Disease (COPD)	✓		
Gout	✓		
Osteoarthritis	✓		
Osteoporosis	✓		

Table S2. Comorbidity Classification Categories

Comorbidity	Category
Cirrhosis	Not identified
	Diagnosed within 365 days of the index date
	Diagnosed more than 365 days before the index date
Hepatitis B Virus (HBV) Infection	Not identified
	HBV without HDV diagnosed within 365 days of the index date
	HBV without HDV diagnosed more than 365 days before the index date
	HBV with HDV diagnosed within 365 days of the index date
	HBV with HDV more than 365 days before the index date
Hepatitis C Virus (HCV) Infection	Not identified
	Diagnosed within 365 days of the index date
	Diagnosed more than 365 days before the index date
Steatosis of Liver	Not identified
	Diagnosed within 365 days of the index date
	Diagnosed more than 365 days before the index date
Autoimmune Liver Disease	Not identified
	Diagnosed within 365 days of the index date
	Diagnosed more than 365 days before the index date
Diabetes	Not identified
	Other diabetes diagnosed within 365 days of the index date
	Other diabetes diagnosed more than 365 days before the index date
	Type 1 diabetes diagnosed within 365 days of the index date
	Type 1 diabetes diagnosed more than 365 days before the index date
	Type 2 diabetes diagnosed within 365 days of the index date
	Type 2 diabetes diagnosed more than 365 days before the index date
	Prediabetes diagnosed within 365 days of the index date

	Prediabetes diagnosed more than 365 days before the index date
Hypertension	Not identified
	Primary hypertension diagnosed within 365 days of the index date
	Primary hypertension diagnosed more than 365 days before the index date
	Secondary hypertension diagnosed within 365 days of the index date
	Secondary hypertension diagnosed more than 365 days before the index date
Hyperlipidemia	Not identified
	Diagnosed within 365 days of the index date
	Diagnosed more than 365 days before the index date
Cholangitis	Not identified
	Other cholangitis within 365 days of the index date
	Other cholangitis diagnosed more than 365 days before the index date
	Primary sclerosing cholangitis diagnosed within 365 days of the index date
	Primary sclerosing cholangitis diagnosed more than 365 days before the index date
Gallstone	Not identified
	Diagnosed within 365 days of the index date
	Diagnosed more than 365 days before the index date
Cholesterolosis of Gallbladder	Not identified
	Diagnosed within 365 days of the index date
	Diagnosed more than 365 days before the index date
Anxiety	Not identified
	Diagnosed within 365 days of the index date
	Diagnosed more than 365 days before the index date
Depressive Disorder	Not identified
	Diagnosed within 365 days of the index date
	Diagnosed more than 365 days before the index date
Gastroesophageal Reflux Disease (GERD)/Peptic Ulcer	Not identified
	GERD diagnosed within 365 days of the index date
	GERD diagnosed more than 365 days before the index date
	Peptic ulcer diagnosed within 365 days of the index date

	Peptic ulcer diagnosed more than 365 days before the index date
	PPI use started within 365 days of the index date
	PPI use started more than 365 days before the index date
Ulcerative Colitis	Not identified
	Diagnosed within 365 days of the index date
	Diagnosed more than 365 days before the index date
Crohn's Disease	Not identified
	Diagnosed within 365 days of the index date
	Diagnosed more than 365 days before the index date
Polyp of Large Intestine	Not identified
	Diagnosed within 365 days of the index date
	Diagnosed more than 365 days before the index date
Chronic Kidney Disease	Not identified
	Diagnosed within 365 days of the index date
	Diagnosed more than 365 days before the index date
Kidney Stone	Not identified
	Diagnosed within 365 days of the index date
	Diagnosed more than 365 days before the index date
Vitamin D Deficiency	Not identified
	Diagnosed within 365 days of the index date
	Diagnosed more than 365 days before the index date
Alcohol Dependence	Not identified
	Diagnosed within 365 days of the index date
	Diagnosed more than 365 days before the index date
Nicotine Dependence	Not identified
	Diagnosed within 365 days of the index date
	Diagnosed more than 365 days before the index date
Hypothyroidism	Not identified
	Diagnosed within 365 days of the index date
	Diagnosed more than 365 days before the index date
Human Immunodeficiency Virus (HIV) Infection	Not identified

	Diagnosed within 365 days of the index date
	Diagnosed more than 365 days before the index date
Asthma	Not identified
	Diagnosed within 365 days of the index date
	Diagnosed more than 365 days before the index date
Obstructive Sleep Apnea (OSA)	Not identified
	Diagnosed within 365 days of the index date
	Diagnosed more than 365 days before the index date
Allergic Rhinitis	Not identified
	Diagnosed within 365 days of the index date
	Diagnosed more than 365 days before the index date
Congenital Heart	Not identified
	Diagnosed within 365 days of the index date
	Diagnosed more than 365 days before the index date
Coronary Arteriosclerosis	Not identified
	Diagnosed within 365 days of the index date
	Diagnosed more than 365 days before the index date
Anemia	Not identified
	Chronic disease related anemia diagnosed more than 365 days before the index date
	Primary anemia diagnosed more than 365 days before the index date
	Other anemia diagnosed more than 365 days before the index date
Gastritis	Not identified
	Diagnosed within 365 days of the index date
	Diagnosed more than 365 days before the index date

Table S3. F1 Score of Early-Onset Liver Cancer and HCC Prediction Models on the Validation Dataset

**Classification method**	**All**	**Asian and Pacific Islander**	**Hispanic**	**White**	**Other/Unknown**
**Early-onset liver cancer**					
Logistic Regression	0.67	0.77	0.75	0.64	0.68
LightGBM	0.65	0.69	0.75	0.60	0.61
XGBoost	0.64	0.61	0.77	0.62	0.66
Random Forest	0.57	0.61	0.76	0.57	0.62
Decision Tree	0.45	0.61	0.74	0.33	0.19
**Early-onset HCC**					
Logistic Regression	0.70	0.81	0.82	0.64	0.81
LightGBM	0.69	0.79	0.82	0.64	0.66
XGBoost	0.67	0.80	0.84	0.64	0.74
Random Forest	0.68	0.80	0.80	0.61	0.74
Decision Tree	0.54	0.73	0.71	0.57	0.66

Note: HCC, Hepatocellular carcinoma.

Table S4. Summary of Machine Learning Model Training and Validation

Component	Details
Platform	Databricks AutoML on AWS
Algorithms Used	Logistic Regression, Decision Trees, Random Forests, XGBoost, LightGBM
Data Splitting	AutoML splits the input dataset into training (60%), validation (20%), and test (20%) sets by default.
Feature Engineering	Automatically detects feature types (numeric, categorical, text) and applies transformations such as one-hot encoding.
Class Imbalance Handling	Automatically detects class imbalance and addresses it by down-sampling the majority class and applying class weights when imbalance is detected.
Hyperparameter Tuning	Databricks AutoML facilitates hyperparameter tuning by integrating distributed optimization libraries such as Optuna and Ray Tune, with MLflow for tracking. This enables scalable and efficient model selection across clusters.
Final Model Selection	AutoML automatically trains multiple models and ranks them based on performance on the validation set using classification metrics. The model with the highest validation F1 score is selected by default for classification tasks.
References	Databricks. What is AutoML? | Databricks Documentation. February 14, 2025. Accessed April 21, 2025. https://docs.databricks.com/aws/en/machine-learning/automl

Table S5. Summary of SHAP (SHapley Additive exPlanations) in the Context of This Study

**Aspect**	**Description**
Mathematical background	Ponce-Bobadilla et al. (2024) proposed a scenario with three drugs—A, B, and C—which can be used alone or in combination to treat a condition. Each drug and drug combination has a known response rate, and the combination of all three drugs leads to a 90% response rate. The question is: how much does each individual drug contribute to that overall response?<br>To answer this, the authors compute the marginal contribution of each drug across all possible combinations of the other drugs. For instance, they compare the response of Drug A combined with Drug B to the response of Drug B alone to measure Drug A’s added value in that setting. This is repeated for all subsets involving A, B, and C.<br>By averaging the contribution of each drug across all combinations, they calculate the Shapley value for each drug, which represents its fair share of the treatment effect. This approach is similar to how SHAP values are used in machine learning to explain the role of different features in a model's predictions.
Model Compatibility	SHAP can be applied to various machine learning algorithms, including tree-based models, linear models, and deep learning models.
Application in Study	In our study, SHAP was utilized to identify and rank the most influential comorbidities associated with liver cancer risk prediction in different racial groups.
References	Ponce- Bobadilla AV, Schmitt V, Maier CS, Mensing S, Stodtmann S. Practical guide to SHAP analysis: Explaining supervised machine learning model predictions in drug development. Clinical and Translational Science. 2024;17(11). doi:10.1111/cts.70056

Figure S2. Race/ethnicity-specific feature importance plots among patients with early-onset HCC. Panel A represents the model for Asian/Pacific Islanders, Panel B for Hispanics, Panel C for Whites, and Panel D for Other/Unknown.

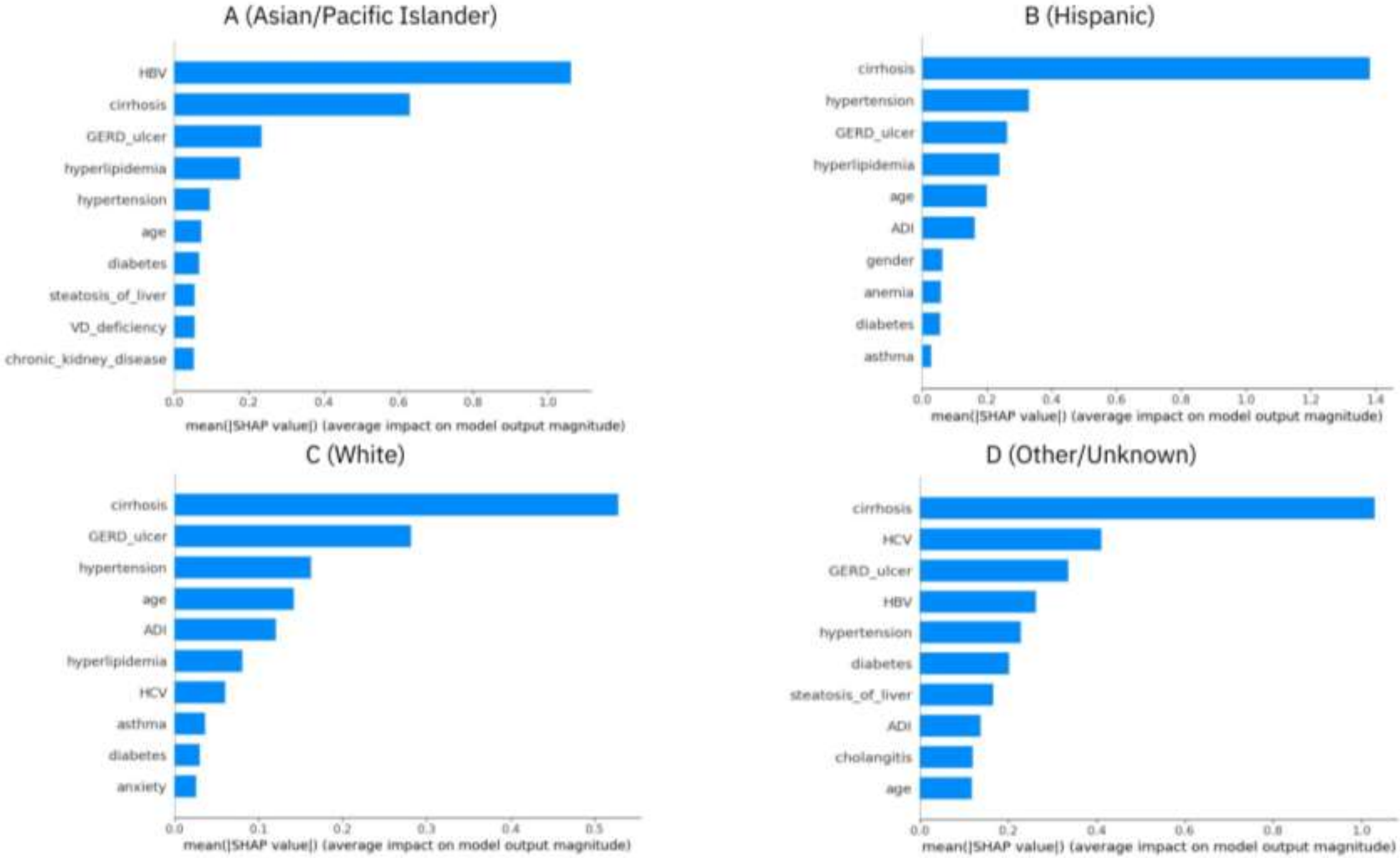

Note: HBV, Hepatitis B Virus; GERD, Gastroesophageal Reflux Disease; VD, Vitamin D; ADI, Area Deprivation Index; HCV, Hepatitis C Virus; SHAP, SHapley Additive exPlanations.
